# Which disease-related factors influence patients’ and physicians’ willingness to consider joint replacement in hip and knee OA? Results of a questionnaire survey linked to claims data

**DOI:** 10.1186/s12891-020-03368-1

**Published:** 2020-06-05

**Authors:** Anne Postler, Jens Goronzy, Klaus-Peter Günther, Toni Lange, Imke Redeker, Jochen Schmitt, Angela Zink, Johanna Callhoff

**Affiliations:** 1 University Center of Orthopaedics and Traumatology, University Medicine Carl, Gustav Carus Dresden, TU Dresden, Fetscherstr. 74, 01307 Dresden, Germany; 2 Epidemiology Unit, German Rheumatism Research Centre, Berlin, Germany; 3 Center for Evidence-Based Healthcare, Medical Faculty, Technical University, Dresden, Dresden, Germany; 4 Department of Gastroenterology, Infectiology and Rheumatology, Charité – Universitätsmedizin Berlin, Berlin, Germany

**Keywords:** Hip or knee osteoarthritis, Conservative treatment in osteoarthritis, Total joint replacement, Health service needs and demands, Patient preferences, National guideline

## Abstract

**Background:**

A great heterogeneity in total joint replacement (TJR) rates has been reported for osteoarthritis (OA), most likely arising from a gap between patients’ and physicians’ views on the need for TJR. The purpose of this study therefore was to analyze potential cofactors which might influence the desire of patients to undergo TJR and physicians’ willingness to discuss surgery with their patients.

**Methods:**

A total of 8995 patients in Germany with a claims data diagnosis of hip or knee OA or polyarthrosis were asked to complete a questionnaire for this cross-sectional study of sociodemographic factors, indicators of current joint function (WOMAC score), willingness to undergo TJR and whether they had already discussed TJR with a physician. The overall response rate was 40%. Responders with polyarthrosis and individuals without current or chronic symptoms in the corresponding joints, pain in already replaced joints or simultaneous symptomatic hip and knee OA were excluded. We linked the survey results to claims data. Separate logistic regression models were used to assess which parameters were associated with patients’ willingness to undergo TJR and physicians’ discussion of surgery.

**Results:**

**We analyzed** 478 hip OA and 932 knee OA patients. Just 17% with hip OA and 14% with knee OA were willing to undergo TJR, although 44 and 45% had already discussed surgery with their physicians.

Patients’ willingness was associated with higher WOMAC scores, a deterioration of symptoms over the last 2 years, and previous TJR for another joint. The discussion with a physician was influenced by the impact on personal life and previous arthroplasty. Older age (odds Ratio (OR) 1.2 per 10 years), male sex (OR 0.69 vs female), longer symptom duration (OR 1.08 per 5 years), deterioration of symptoms (OR 2.0 vs no change/improvement), a higher WOMAC score (OR 1.3 per 10% deterioration) and reduced well-being (OR 1.1 per 10% deterioration) were associated with physician discussion in knee OA patients.

**Conclusions:**

The proportion of patients willing to undergo TJR is lower than the proportion in whom physicians discuss surgery. While previous TJR seems to enhance patients’ and surgeons’ willingness, the influence of other cofactors is heterogeneous.

## Background

Osteoarthritis (OA) is the most prevalent chronic joint disease in the world. Half of the world’s population aged 65 and older suffers from some form of OA [1]. It is one of the most common sources of pain and disability in the elderly [1–3], and international reports indicate that the prevalence of OA is on the rise [4]. Stepped-care strategies include recommendations for non-surgical treatment of hip and knee OA [5–8]. Failed conservative therapy, pain, loss of function and radiological changes are generally considered in the decision to perform total hip replacement (THR) or total knee replacement (TKR) [9, 10]. Nevertheless, the decision when to perform total joint replacement (TJR) is not well defined. As a result, there is great variation both in indications for surgery among orthopaedic surgeons [11, 12] and in the utilization of arthroplasty in general [13].

In addition to surgeon recommendations, patient preferences also play an important role in the decision process. In advanced OA, as with other disorders for which multiple treatments are available, shared decision-making helps patients and physicians to choose the treatment that best fits a patient’s preference [14]. Several studies investigating the interrelationship between surgeons’ recommendations and patients’ willingness to undergo TJR report a wide gap between the views of both partners in this decision-making process [15–18]. Patients’ perceptions of the appropriateness of and their desire for TJR are of substantial importance in the discussion of treatment alternatives [12] and have to be appropriately included in shared decision-making [10]. Although several international studies have investigated factors potentially influencing patients’ decisions to undergo surgery [12, 19–21], it is still unclear why patients’ perspectives often differ from surgeons’ recommendations. The aim of our study, therefore, is to investigate the influence of disease-related cofactors on the willingness of patients and the advice of surgeons to consider TJR in a large population of insured individuals with hip or knee OA.

## Methods

### Participants

This study is part of the PROCLAIR (“Linking **P**atient-**R**eported **O**utcomes with **CLAI**ms data for health services **R**esearch in rheumatology“) collaborative project. In the setting of this project, we used data from one of the largest German statutory health insurers (BARMER, > 9 million beneficiaries in 2018) to identify patients with OA of the knee or hip or polyarthritis (ICD-10-GM Codes M15–17) in at least two quarters in 2014. Germany has a mandatory health insurance system for all citizens, of which a minority is covered by private health insurance and the majority by statutory health insurance providers (more than 90%), of which BARMER is a major one. The study used stratified sampling, and each stratum was sampled from patients who were continuously insured with BARMER in 2014 and 2015. We stratified for age (30–39, 40–49, 50–59, 60–69, 70–79), sex and diagnosis (M15: polyarthritis, M16: OA of the hip, M17: OA of the knee). With one exception, each stratum included 330 persons. In the stratum of men with polyarthritis aged 30–39 years, there were only 164 individuals who were all selected. The total sample size (*n* = 9734) was planned in order to detect differences of 8% in prescription frequency (e.g., physical therapy) in the total population and in subgroups such as knee OA or hip OA.

Inclusion criteria were

-Continuous insurance with BARMER from 2014 to 2016

-Having a claims diagnosis of OA (ICD-10-GM Codes M16/M17) in at least two quarters of 2014

-Written informed consent to the linking of survey results and claims data

-Self-reported acute or chronic symptoms in either the hip or knee

Exclusion criteria were

-Having a claims diagnosis of M15

-Having a claims diagnosis of both M16 and M17 and self-reported current or chronic symptoms in the hip and knee

-No longer insured with BARMER when the survey was conducted in 2016

**Fig. 1:**
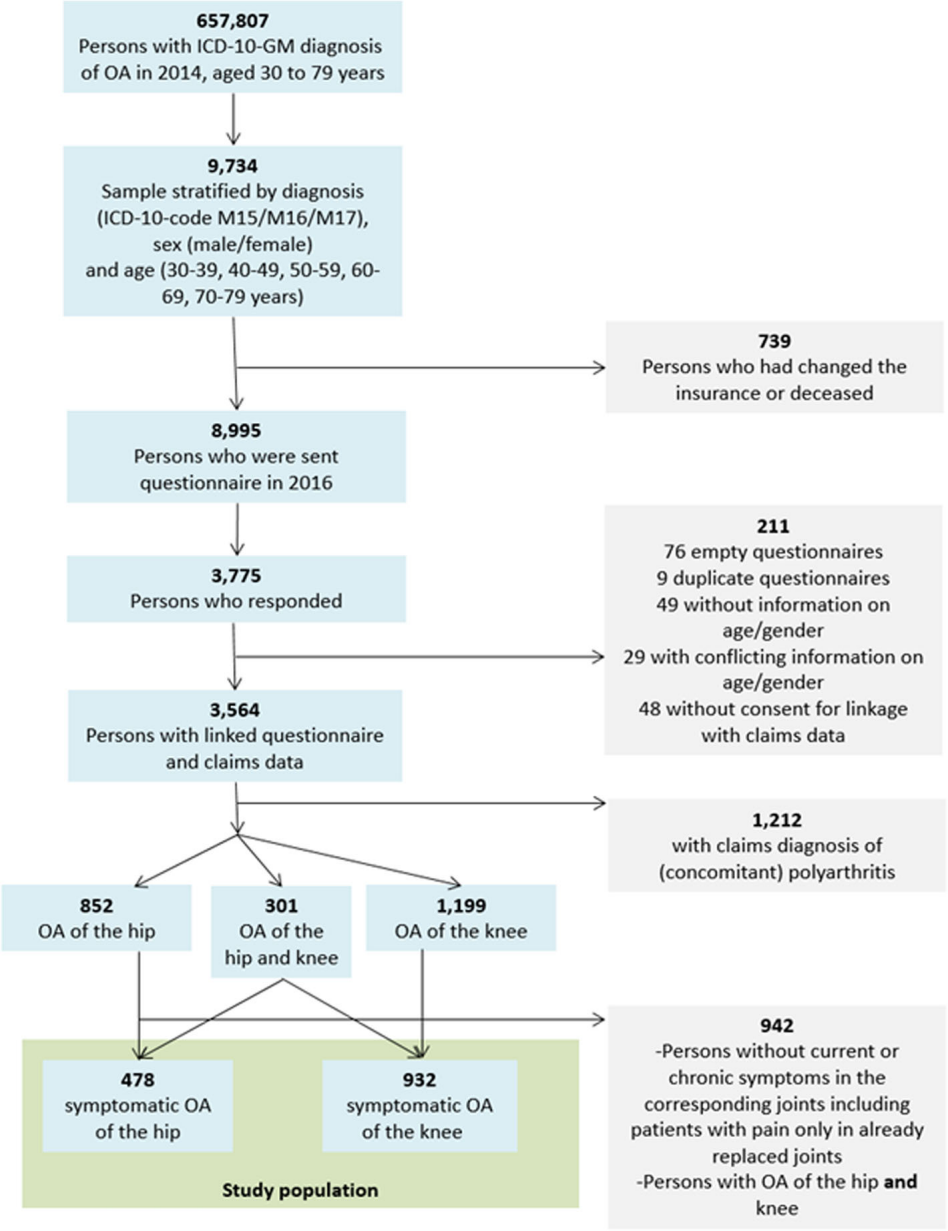
Flowchart of participants and selection process. ICD-10-GM: International classification of diseases, 10th revision, German modification, OA: Osteoarthritis

Figure 1 provides a flowchart of the selection process including how many persons were excluded at each stages.

The data for analysis were obtained from two sources: results of a questionnaire survey (see attachment) conducted in 2016 and insurance claims data.

After exclusion of individuals who changed their health insurance or died in the study period from 2014 to 2016, we mailed questionnaires and one reminder to 8995 persons in the summer of 2016.

### Data collection

The questionnaire used in our survey covered the following domains: painful joints during the last 7 days or during at least 3 months in the last 2 years, symptom duration, diagnostic procedures used (X-ray, MRI), physician mainly treating the OA (orthopaedic specialist, rheumatologist, general practitioner or other specialist), health-related quality of life using the Western Ontario and McMaster Universities Osteoarthritis Index, WOMAC [22, 23], the WHO-5 index of well-being [24, 25], the impact of the OA on the personal and professional life and sociodemographic variables. The WOMAC and its sub-scores for pain, stiffness and physical function are expressed as percentages with 100 representing the worst outcome. The WHO-5 index measures well-being on a scale of 0 (worst outcome) to 100 (best outcome). In addition, the questionnaire asked about a history of prior TJR surgery (any joint) in order to exclude patients with pain only in already replaced joints). Finally, participants were asked if their mainly treating physician had already discussed the possibility of TJR and whether they themselves were currently be willing to undergo TJR.

The patients who completed and returned the questionnaires in 2016 were asked for written informed consent to the linking of their questionnaire data with claims data. Prescriptions of physical therapy was collected and prescriptions of analgesics were identified using ATC (Anatomical Therapeutic Chemical Classification System) codes. For each medication, a prescription was assumed when the participant received at least one prescription for that drug.

As an indicator of comorbidity in the surveyed patients, we calculated the number of different medication prescriptions for drugs other than analgesics such as opioids or NSAIDs. The number of prescribed medications was recorded to quarters. Furthermore, we used the Elixhauser index, which was developed for studies using large administrative databases and includes 31 severe comorbidities [26] other than OA of the hip (M16) or knee (M17).

### Statistical analysis

The results were weighted with respect to the distribution of all participants with OA in the claims data. Subgroup analysis for persons with hip or knee OA was conducted with the domain analysis tool using procedures for complex survey samples in the SAS/STAT® software package (Version 9.4, Copyright© 2013 SAS Institute Inc.).

We checked for non-responder bias in the questionnaire survey by comparing age, sex, the number of medication prescriptions, treating physician (orthopaedic or other specialist) and conservative treatment (opioids, NSAIDs or physical therapy) was prescribed between responders and non-responders.

Logistic regression models were used to analyze which factors were associated with a desire for TJR and with having discussed TJR. Multiple imputations with 20 imputations were used for all variables in the models. The association of age and sex with the willingness to undergo TJR and having discussed a TJR with the mainly treating physician was explored. We decided to separately investigate associations between several proxy measures of disease severity (WOMAC, impact on personal life, treatment) and outcomes, adjusting for age and sex as possible confounders. The different measures of disease severity are highly correlated. In this cross-sectional analysis, it was not possible to correct for confounding by indication.

Ethical approval for collaborative project PROCLAIR was obtained from the ethics committee of Charité - Universitätsmedizin in March of 2015 (EA1/051/15) and was conducted in agreement with the Declaration of Helsinki.

## Results

The characteristics of the study population are summarized in Fig. 1 and Table 1.

**Table 1:** Characteristics of the study population

Variable		Missing values	Hip OA ***n*** = 478	Knee OA ***n*** = 932
Demographics	Age (years), mean	0	64 (63; 65)	65 (64; 65)
	Female	0	63 (58; 67)	68 (66; 70)
	BMI	29	28 (27; 28)	29 (29; 30)
Burden of OA	Symptom duration (years), mean	72	14 (12; 15)	15 (14; 16)
	Deterioration of symptoms (during last 2 years)	21	62 (56; 68)	57 (53; 61)
WOMAC	WOMAC total, mean	202	42 (39; 44)	41 (39; 42)
	WOMAC pain, mean	131	42 (40; 45)	42 (40; 43)
	WOMAC function, mean	89	41 (38; 43)	39 (38; 41)
	WOMAC stiffness, mean	89	45 (42; 48)	45 (43; 47)
Any impact on personal situation		27	78 (73; 83)	77 (74; 81)
WHO-5		61	50 (47; 53)	51 (49; 53)
Treating specialist	Orthopaedist is treating	12	63 (57; 69)	71 (68; 75)
Diagnostics	X-Ray of the corresponding joint	15	92 (89; 95)	91 (89; 93)
	MRI of the corresponding joint	49	42 (36; 48)	55 (51; 59)
Treatment	No prescription of analgesics^a^	0	42 (36; 48)	34 (31; 38)
	NSAR prescription^a^	0	44 (38; 50)	51 (47; 55)
	Opioid prescription^a^	0	13 (9; 18)	13 (10; 16)
	Other analgetic prescription^a^	0	25 (20; 30)	29 (26; 33)
	Daily use of medication	20	26 (21; 31)	22 (19; 25)
	Occasional use of medication	20	46 (40; 52)	51 (47; 55)
	Physical therapy prescription^a^	0	46 (40; 52)	49 (45; 53)
	Having at least one artificial joint	52	23 (18; 28)	19 (16; 22)

Values are percentages unless indicated otherwise. Values in brackets are 95% confidence intervals. ^a^ indicates information derived from claims data

A total of 3775 patients completed and returned the questionnaires, and 478 patients with symptomatic hip OA and 932 with symptomatic knee OA were analyzed. Mean age was 64 years (95% confidence interval (CI) 63–65) in the group with hip OA and 65 years (95% CI 64–65) in the group with knee OA.

Mean symptom duration was 14 years (95% CI 12–15) for hip OA and 15 years (14–16) for knee OA. The total WOMAC score was 42 (95% CI 39–44) in hip OA and 41 (95% CI 39–42) in knee OA.

An orthopaedic specialist) was reported as the mainly treating physician by 63% (hip OA) and 71% (knee OA) of the patients. Physical therapy was prescribed in 46% (hip OA) and 49% (knee OA), whereas any kind of analgesic was prescribed in 58 and 66%, respectively.

Individuals who returned the completed questionnaire were slightly older (67.2 vs. 65.5 years), and more of them were seeing an orthopaedic specialist (54% vs. 44%) or had a prescription of NSAIDs (48% vs. 41%) than those who did not answer the questionnaire. The percentage of women, Elixhauser comorbidity index (median 2.5 vs. 2.7), and prescriptions of opioids (13.8% vs. 13.5%) did not differ clinically meaningful between responders and non-responders.

Overall, 17% of the patients with hip OA were willing to undergo TJR while 83% stated they were not. The distribution was similar for knee OA (Table 2). Most of the patients who had discussed TJR had done so with an orthopaedic specialist (83% hip OA, 86% knee OA). In the group willing to undergo TJR, patients had higher WOMAC scores, more patients reported deterioration of their symptoms over the last 2 years before the survey or an impact on personal life, and there were more patients with previous arthroplasty of other joints and treatment by an orthopaedist compared with the group of patients who were unwilling (Table 3). We performed logistic regression adjusted for age, sex and symptom duration to determine factors associated with the willingness to undergo TJR (Table 4). For both hip and knee OA, it was associated with reduced joint function, as reflected by a high WOMAC score (OR hip 1.47, 95% CI 1.24–1.75; OR knee 1.42, 95% CI 1.26–1.61), a greater impact on personal life (OR hip 10.3, 95% CI 1.04–103; OR knee 6.81, 95% CI 2.23–20.79), worsening of symptoms over the last 2 years (OR hip 2.73, 95% CI 1.14–6.55; OR knee 3.66, 95% CI 2.05–6.53) and already having one artificial joint (OR hip 6.01, 95% CI 3–12.06; OR knee 3.69, 95% CI 2.2–6.18). For knee OA, decreased well-being was associated with a greater willingness to undergo TJR while for hip OA alone, only treatment by an orthopaedic specialist had an effect on the patient’s wish to undergo TJR.

**Table 2:** Comparison of patients’ willingness and physicians’ discussion of TJR in hip / knee OA. All values are percentages

	Patients’ willingness to undergo TJR		
Physicians’ discussion of TJR	Yes	No	Total
*Hip OA*			
**Yes**	14.2	29.6	43.7
**No**	2.8	53.4	56.2
Total	17.0	83.0	
*Knee OA*			
**Yes**	12.5	32.7	45.2
**No**	1.5	53.3	54.8
Total	14.0	86.0

**Table 3:** Distribution of survey results and claims data in patient groups willing and unwilling to undergo TJR

	Symptomatic Hip OA		Symptomatic Knee OA	
Variable	Willing for TJR ***n*** = 79, 17% (weighted)	Unwilling for TJR ***n*** = 388, 83% (weighted)	Willing for TJR ***n*** = 127 14% (weighted)	Unwilling for TJR ***n*** = 783 86% (weighted)
Age (years), mean	63 (61; 66)	65 (64; 66)	65 (64; 67)	65 (64; 65)
Female	63 (50; 76)	63 (58; 68)	63 (54; 72)	68 (66; 71)
BMI (kg/m^2^), mean	27 (26; 28)	28 (27; 28)	30 (29; 30)	29 (29; 30)
BMI < =18.5	. (.;.)	2 (0; 5)	. (.;.)	1 (0; 1)
18.5 < BMI < =25	37 (22; 52)	31 (25; 38)	14 (6; 21)	21 (17; 25)
25 < BMI < =30	43 (29; 57)	41 (34; 48)	49 (38; 59)	41 (37; 45)
BMI > 30	20 (10; 30)	26 (20; 32)	38 (27; 48)	37 (33; 41)
Symptom duration (years), mean	14 (10; 18)	14 (12; 16)	17 (14; 19)	14 (13; 15)
Deterioration of symptoms (during last 2 years)	80 (67; 93)	59 (53; 66)	80 (72; 89)	53 (49; 57)
**WOMAC total**, mean	52 (46; 57)	40 (37; 42)	52 (49; 56)	39 (37; 41)
WOMAC pain, mean	51 (45; 57)	41 (38; 43)	53 (50; 56)	40 (38; 42)
WOMAC function, mean	51 (46; 57)	38 (36; 41)	51 (48; 55)	37 (36; 39)
WOMAC stiffness, mean	52 (45; 60)	43 (40; 46)	57 (53; 61)	43 (41; 45)
Any impact on personal situation	99 (96; 100)	75 (69; 81)	95 (90; 100)	75 (71; 79)
WHO-5	49 (42; 56)	51 (47; 54)	44 (39; 49)	52 (50; 54)
Orthopaedist is treating	78 (65; 92)	60 (53; 67)	78 (69; 86)	71 (67; 75)
Opioid prescription^a^	13 (2; 23)	14 (9; 19)	22 (13; 30)	12 (9; 15)
Daily use of medication	28 (15; 41)	26 (20; 32)	37 (27; 47)	20 (17; 24)
Physical therapy prescription^a^	50 (35; 65)	45 (38; 52)	54 (43; 64)	47 (43; 51)
Having at least one artificial joint	53 (38; 67)	17 (12; 23)	41 (31; 52)	16 (13; 19)

Values are percentages unless indicated otherwise. Values in brackets are 95% confidence intervals. ^a^ indicates information derived from claims data

**Table 4:** Results of logistic regression models – factors associated with **patients’ willingness** to undergo TJR

Variable	Reference	OR hip	95% CI hip	***p***-Value hip	OR knee	95% CI knee	***p-***Value knee
Age, years	per 10-year increment	0.9	(0.67; 1.19)	0.45	1.09	(0.9; 1.31)	0.38
Sex, female	Male	0.99	(0.53; 1.86)	0.98	0.77	(0.5; 1.18)	0.22
BMI > =35 kg/m^2a^	< 35 kg/m^2^	0.44	(0.15; 1.36)	0.15	1	(0.53; 1.91)	1
Symptom duration, years^b^	per 5-year increment	1.01	(0.86; 1.19)	0.9	1.07	(0.99; 1.16)	0.09
Deterioration of symptoms over the 2 preceding years^a^	no change/ improvement	**2.73**	**(1.14; 6.55)**	**0.02**	**3.66**	**(2.05; 6.53)**	**< 0.01**
WOMAC^a^	per 10-unit increase	**1.47**	**(1.24; 1.75)**	**< 0.01**	**1.42**	**(1.26; 1.61)**	**< 0.01**
Any impact on personal life^a^	No impact	**10.3**^**c**^	**(1.04; 103)**	**0.05**	**6.81**	**(2.23; 20.79)**	**< 0.01**
WHO-5^a^	per 10 units increase	0.98	(0.86; 1.11)	0.72	**0.86**	**(0.79; 0.95)**	**< 0.01**
Treatment by orthopaedist ^a^	Treatment by other physician	**2.39**	**(1.04; 5.48)**	**0.04**	1.44	(0.84; 2.46)	0.18
NSAID prescription^a^	No	0.97	(0.53; 1.77)	0.91	1.27	(0.78; 2.07)	0.34
Opioid prescription^a^	No	0.9	(0.32; 2.54)	0.85	1.83	(1.01; 3.34)	0.05
Physical therapy prescription^a^	No	1.24	(0.65; 2.39)	0.51	1.19	(0.72; 1.95)	0.49
Having at least one artificial joint	No	**6.01**	**(3; 12.06)**	**< 0.01**	**3.69**	**(2.2; 6.18)**	**< 0.01**

^a^adjusted for age, sex, symptom duration

^b^adjusted for age, sex

^c^This result is not robust (see confidence interval) since there are only few patients willing for TJR without impact on their personal life

Results of 11 multiple logistic regression models with “patients’ willingness to undergo TJR” as independent variable. Bold numbers indicate statistically significant associations

Multiple imputation (*n* = 20 imputations) was used to deal with missing values

Regarding the interaction with their mainly treating physician, 44% of hip OA and 45% of knee OA patients had already discussed a possible TJR. However, the majority of patients who reported a previous discussion with their physician (68% hip OA, 72% in knee OA) were not willing to undergo TJR (Table 2). The unadjusted data (Table 5) show certain differences between hip OA and knee OA patient groups regarding the effect of prior discussion of TJR.

**Table 5:** Distribution of survey results and claims data in patient groups with and without prior discussion of the option of TJR by physician

	Symptomatic Hip OA		Symptomatic Knee OA	
Variable	TJR discussed ***n*** = 215, 44% (weighted)	TJR not discussed ***n*** = 255, 56% (weighted)	TJR discussed ***n*** = 387, 45% (weighted)	TJR not discussed ***n*** = 524, 55% (weighted)
Age (years), mean	64 (62; 65)	65 (63; 66)	66 (65; 67)	64 (63; 65)
Female	61 (53; 68)	64 (57; 70)	64 (59; 68)	71 (68; 74)
BMI > 30 (kg/m^2^)	24 (17; 31)	26 (19; 33)	35 (29; 40)	39 (33; 44)
Symptom duration (years), mean	14 (12; 16)	14 (12; 16)	16 (14; 17)	13 (12; 15)
Deterioration of symptoms (during last 2 years)	64 (55; 73)	62 (54; 70)	67 (61; 72)	50 (44; 55)
**WOMAC total**, mean	43 (39; 46)	41 (38; 44)	46 (44; 49)	36 (34; 38)
WOMAC pain, mean	42 (38; 45)	43 (39; 46)	47 (45; 49)	37 (35; 40)
WOMAC function, mean	42 (38; 45)	40 (36; 43)	45 (42; 47)	35 (32; 37)
WOMAC stiffness, mean	44 (40; 49)	45 (42; 49)	50 (47; 53)	40 (38; 43)
Any impact on personal situation	89 (83; 94)	72 (64; 79)	85 (80; 89)	72 (67; 77)
WHO-5	51 (47; 56)	50 (46; 54)	48 (45; 51)	53 (50; 56)
Orthopaedist is treating	68 (60; 77)	58 (50; 67)	77 (72; 82)	68 (63; 73)
Opioid prescription^a^	14 (7; 20)	13 (8; 19)	17 (12; 21)	10 (7; 13)
Daily use of medication	24 (16; 32)	28 (20; 35)	27 (22; 33)	18 (14; 22)
Physical therapy prescription^a^	46 (37; 55)	45 (37; 54)	51 (45; 57)	47 (42; 52)
Patient desiring TJR: knee or hip	32 (24; 41)	5 (2; 8)	28 (22; 33)	3 (1; 5)
Having at least one artificial joint	36 (27; 45)	13 (7; 20)	29 (24; 34)	11 (7; 14)

Values are percentages unless indicated otherwise. Values in brackets are 95% confidence intervals. ^a^ indicates information derived from claims data

Among patients who had already discussed TJR with their physician, we searched for factors associated with willing to undergo TJR. In this subgroup, both knee and hip OA patients who were willing to under TJR had poorer joint function than those who were not willing (WOMAC score of 52 vs. 44 for hip OA and 50 vs. 39 for knee).

We fitted logistic regression models to identify factors associated with having discussed TJR among all hip and knee OA patients. The models were adjusted for age, sex and symptom duration (Table 6). In both hip and knee OA patients, significant associations with physician discussion were only found for impact on personal life and already having received another TJR. Older age, male sex, longer symptom duration, deterioration of symptoms over the last 2 years before the survey, a higher WOMAC score and reduced well-being were associated with having discussed TJR in knee OA, but not in hip OA.

**Table 6:** Results of logistic regression models – factors associated with having **discussed** TJR

Variable	Reference	OR hip	95% CI hip	***p-***Value hip	OR knee	95% CI knee	***p-***Value knee
Age, years	per 10 years increase	0.9	(0.72; 1.12)	0.35	**1.23**	**(1.07; 1.42)**	**< 0.01**
Sex female	Male	0.87	(0.54; 1.41)	0.58	**0.69**	**(0.51; 0.94)**	**0.02**
BMI > =35 kg/m^2a^	< 35 kg/m^2^	0.45	(0.18; 1.11)	0.08	1.21	(0.75; 1.95)	0.44
Symptom duration, years^b^	per 5 years increase	1	(0.9; 1.12)	0.96	**1.08**	**(1; 1.15)**	**0.04**
Deterioration of symptoms in last 2 years^a^	no change/improvement	1.1	(0.66; 1.84)	0.72	**2.02**	**(1.44; 2.84)**	**< 0.01**
WOMAC^a^	per 10 units increase	1.04	(0.91; 1.18)	0.57	**1.29**	**(1.17; 1.42)**	**< 0.01**
Any impact on personal life^a^	No impact	**2.65**	**(1.29; 5.46)**	**0.01**	**2.32**	**(1.5; 3.6)**	**< 0.01**
WHO-5^a^	per 10 units increase	1.03	(0.93; 1.15)	0.59	**0.91**	**(0.85; 0.97)**	**0.01**
Orthopaedist is treating^a^	Other physician is treating	1.54	(0.9; 2.62)	0.11	**1.58**	**(1.1; 2.28)**	**0.01**
NSAID prescription^a^	No	1.39	(0.84; 2.3)	0.20	1.3	(0.93; 1.82)	0.13
Opioid prescription^a^	No	0.99	(0.46; 2.15)	0.98	1.64	(0.99; 2.7)	0.05
Physical therapy prescription^a^	No	0.99	(0.6; 1.64)	0.97	1.08	(0.77; 1.52)	0.66
Already having one artificial joint	No	**4.19**	**(2.1; 8.33)**	**< 0.01**	**3.3**	**(2.06; 5.28)**	**< 0.01**

^a^adjusted for age, sex, symptom duration

^b^adjusted for age, sex

Results of 12 multiple logistic regression models with “TJR was discussed” as independent variable. Bold values indicate statistically significant associations

No significant association was found between the number of prescribed medications as an indicator of comorbidity or prescription of physical therapy and discussion of TJR.

## Discussion

Hip and knee replacement is among the medical and surgical interventions with the highest cost effectiveness and capacity to improve patients’ quality of life [27, 28]. Nevertheless, many studies have shown that even patients with advanced OA who are good candidates for TJR are often unwilling to consider surgery [20, 29]. This is confirmed by our findings in a large unselected cohort of insured individuals with the diagnosis of OA. Patients with both hip and knee OA showed little willingness to consider arthroplasty (17 and 14%, respectively). Although not all participants of our study had end-stage OA, a significant disease burden can be assumed since many patients reported a long history of symptoms, worsening over the last 2 years and a high impact on their personal situation. This apparent contradiction between burden and willingness to undergo TJR is what Hudak et al. termed “unmet need” [30]. They found that just a small proportion of patients who were identified as perfect candidates for surgery were actually willing to undergo TJR.

In view of this situation, the question arises: what may induce patients to contemplate surgery as a suitable treatment option? According to Dieppe et al. [31] the decision about TJR is a “judgment call that has to be made by the physician and patient working together, and which has to take account of a large range of complex psychological, social and other issues, in addition to pain, disability and X-ray changes”. They propose a capacity-to-benefit concept consists of interacting disease-related factors (state of the joint, impact on the individual) and treatment-related factors (risk-benefit of TJR and alternative treatment options). This inspired us to analyze the relevance of these possible influencing factors for patients contemplating the need for TJR following many years of conservative treatment of OA.

In both groups - hip and knee OA - we found that patients with poorer joint function (high WOMAC score), deterioration of symptoms over the last 2 years before the survey and impact on personal life were more readily willing to consider TJR. This is in accordance with previous studies describing disease burden as a major driver for the willingness to undergo surgery [12, 29, 32, 33]. A major factor with an impact on the decision about surgery, for both hip and knee OA patients in our study, is the experience with previous TJR of another joint. Although this result reflects common clinical experience in that patients often proceed with additional TJR of other affected joints more readily once they have overcome the barrier of their first decision, it has not been systematically addressed in studies before. Age and sex had not influence the willingness of patients to consider surgery in our study. This is in contrast to earlier investigations [29, 34, 35] reporting associations between demographic factors and the decision for TJR. Another factor not appearing to have had an impact on patients’ attitudes towards surgery in our study is conservative therapy. This result is difficult to interpret, as the proportion of patients with daily intake of analgesics and even opioids was rather low and only about half of the patients had a prescription of physical therapy.

An interesting finding of our study is that, although most patients were treated by an orthopaedic specialist, their counselling led to heterogeneous effects. While patients’ decisions to consider TJR were associated with orthopaedic treatment in hip OA only, significantly more orthopaedists had discussed surgery with knee OA patients than with hip OA patients. As we had no direct access to physicians’ perspectives or to any information objectively documenting the discussions between physicians and their patients, it is impossible to interpret this finding. Nevertheless, the role of physicians in the decision process is important and several studies have highlighted the expectations and attitudes of surgeons and other physicians when proposing TJR to their patients [10, 11, 16, 17, 32, 36, 37]. The most important predictor for patients’ decisions in the study of Hawker et al. [20] was having previously discussed the surgical procedure with a physician.

Our interest was to assess the association of orthopaedist counselling with disease-related cofactors. According to the literature, physicians’ recommendations of TJR should be based on failed conservative therapy, pain, loss of function and radiological changes [9, 10]. As we had no data on radiographic OA grading in our patient population, we focused on prior and current treatment and subjectively perceived disease burden. Neither in patients with hip OA nor in patients with knee OA could we find an association between physicians’ discussion of TJR and prescription of physical therapy or analgesics. This observation confirms the recently described lack of adherence to conservative treatment recommendations in our total cohort of patients with OA in a large German statutory health insurance fund [38, 39]. Regarding the recommendation to base an indication for surgery on pain and functional impairment, we found an association between physicians’ discussion and symptoms (in terms of both duration and deterioration) as well as poorer WOMAC scores and impact on personal life at least in patients with knee OA. In patients with hip OA, however, we only found an association with an impact on patients’ personal life. These results suggest that adherence to treatment guidelines in hip OA is even worse than in knee OA and underline the need for implementing not only already established guidelines for TKR in Germany [10] but also for indication to THR. We also found a high impact of a previous history of TJR at another site on physicians’ discussions of arthroplasty in hip as well as knee OA patients. A positive experience with earlier TJR not only increases patients’ willingness to consider further surgery but also the readiness of physicians to mention this treatment alternative.

Physician support is crucial in the process of shared decision-making. The steps which patients are going through in deciding about arthroplasty include mental preparation, addressing concerns around pain, quality of life and social isolation, weighing risks and benefits, facing fear and uncertainty and the question when “the time is right” for surgery [19]. The consultation is the central part in the preparation of joint replacement surgery. It is where risks and benefits are discussed, trust established, and decisions made [14, 40]. A clear positioning of consulting physicians, whether there is a medical indication, would be helpful in this process. However, published studies suggest that general practitioners, orthopaedists and surgeons differ widely in their attitudes regarding the appropriateness of TJR [32, 36, 37]. Surgeons and referring physicians differ in their opinion about the benefits and timing of surgery [36]. A recent study investigating the influence of patient characteristics on physicians’ and surgeons’ decisions to refer patients to or to perform TKR confirms this variability and also reveals a significant attribution to individual unreliability over time [37]. This inconsistency may enlarge an already existing gap between patients’ and physicians’ perception of the need for surgery and patients’ preferences. This gap can make patients feel anxious and discontent [40], thus contributing to their unwillingness to undergo surgery.

### Limitations and strengths

Our study has several limitations. First, patient selection was based on claims diagnoses of OA and patient-reported symptoms in the corresponding joints. Clinical findings and radiographic grading of OA were not available. However, we believe that the combination of physicians’ diagnoses as documented in claims data and survey-based patient information is a valid measure of disease burden. In addition, the combination of claims and self-reported data ensured that there was no recall bias regarding medication or physical therapy. Second, the results may be biased as patients who returned the questionnaire were likely to be more severely affected than those who did not respond. This non-response bias probably led to worse outcomes in the reported data. Another limitation of our study is that we only asked patients if they had discussed arthroplasty as a potential treatment option with a physician, while we have no direct information from physicians. Therefore, we do not know whether physicians were in favour or against surgery. Nor do we know how well patients remembered the discussion in terms of these issues. It is unclear to what extent this potential drawback may have affected our results. Although we have no information about direction / intensity of advice and timing (early / late or once only / repeated discussion in the disease process), this may not have a substantial impact on our results, as we tried to assess influencing factors on the performed discussion in general. Finally, we did not assess the influence of socioeconomic status and education on patients’ or physicians’ attitude, which are known to affect both willingness and referral patterns [29]. Apart from age, sex and BMI we, however, wanted to investigate explicitly the relevance of disease related factors in the decision process.

A strength of our study is the evaluation of patients who were still in routine care and had not yet been put on a waiting list for TJR. Many earlier studies investigating patients’ and surgeons’ expectations regarding TJR and patients’ willingness to undergo the procedure were performed in preselected patients already scheduled for TJR [15–18]. Other strengths are that we included patients with hip and knee OA as well as patients’ and physicians’ attitudes in our survey, which allows a direct comparison between these important entities. Many studies in the past have only focused on either patient preferences (even separated in hip or knee OA cohorts) or surgeon recommendations. From our point of view it is important to discuss these attitudes and their interrelationship altogether. To our knowledge, this is the first study investigating patients’ willingness compared to physicians’ view to undergo TJR including unselected OA patients in Germany.

## Conclusions

Our results, which are based on a combination of claims data and self-reported patient data, confirm previous reports that the percentage of patients who are willing to undergo TJR is lower than the proportion of patients recommended to have surgery by their physicians. While previous TJR seems to enhance patient’s and surgeons’ willingness, no consistent picture emerges regarding the influence of other cofactors. Especially for patients with hip OA, our results do not reflect internationally established indication criteria for replacement surgery.

## Supplementary information

**Additional file 1.**

## Data Availability

The datasets generated during and analyzed during the current study are available from the corresponding author on reasonable request.

## Abbreviations

ATC: Anatomical-Therapeutic-Chemical Classification System
BMI: Body mass index
CI: Confidence interval
ICD-10: International classification of diseases
MRI: Magnetic resonance imaging
NSAIDs: Non-steroidal anti-inflammatory drugs
OA: Osteoarthritis
OR: Odds ratio
PROCLAIR: Linking **P**atient **R**eported **O**utcomes with **CLAI**ms data for health service **R**esearch in rheumatology
THR: Total hip replacement
TKR: Total knee replacement
TJR: Total joint replacement
WOMAC: Western Ontario and McMaster Universities Osteoarthritis Index
vs.: Versus
