# Supplementary material for: Which disease-related factors influence patients’ and physicians’ willingness to consider joint replacement in hip and knee OA? Results of a questionnaire survey linked to claims data
Source: BMC Musculoskelet Disord. 2020 Jun 5;21:352. doi: 10.1186/s12891-020-03368-1 (PMC7275466; doi:10.1186/s12891-020-03368-1)
Supplement: Supplementary file 1 — Additional file 1. [file 12891_2020_3368_MOESM1_ESM.pptx]

## Slide 1
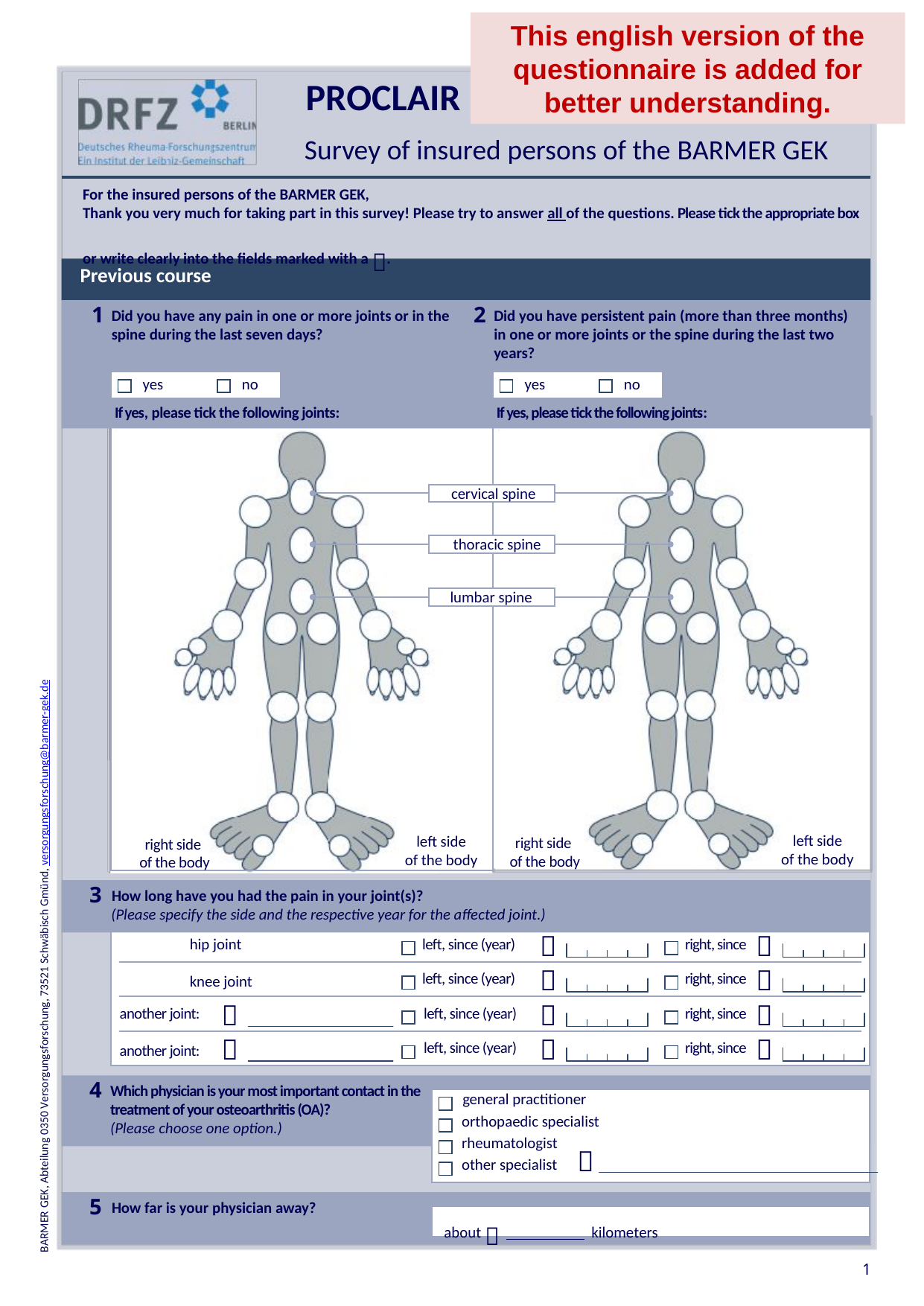

This english version of the questionnaire is added for better understanding.
PROCLAIR
Survey of insured persons of the BARMER GEK
For the insured persons of the BARMER GEK,
Thank you very much for taking part in this survey! Please try to answer all of the questions. Please tick the appropriate box
or write clearly into the fields marked with a .
Previous course
1	2
Did you have any pain in one or more joints or in the
spine during the last seven days?
Did you have persistent pain (more than three months)
in one or more joints or the spine during the last two
years?
yes	no	yes	no
If yes, please tick the following joints:	If yes, please tick the following joints:
cervical spine
thoracic spine
lumbar spine
left side
of the body
left side
of the body
right side
of the body
right side
of the body
3
How long have you had the pain in your joint(s)?
(Please specify the side and the respective year for the affected joint.)








left, since (year)
right, since
right, since
right, since
right, since
hip joint
knee joint
BARMER GEK, Abteilung 0350 Versorgungsforschung, 73521 Schwäbisch Gmünd, versorgungsforschung@barmer-gek.de
left, since (year)


another joint:
another joint:
left, since (year)
left, since (year)
4
Which physician is your most important contact in the
treatment of your osteoarthritis (OA)?
(Please choose one option.)
general practitioner
orthopaedic specialist
rheumatologist

other specialist
5
about  kilometers
How far is your physician away?
1

## Slide 2
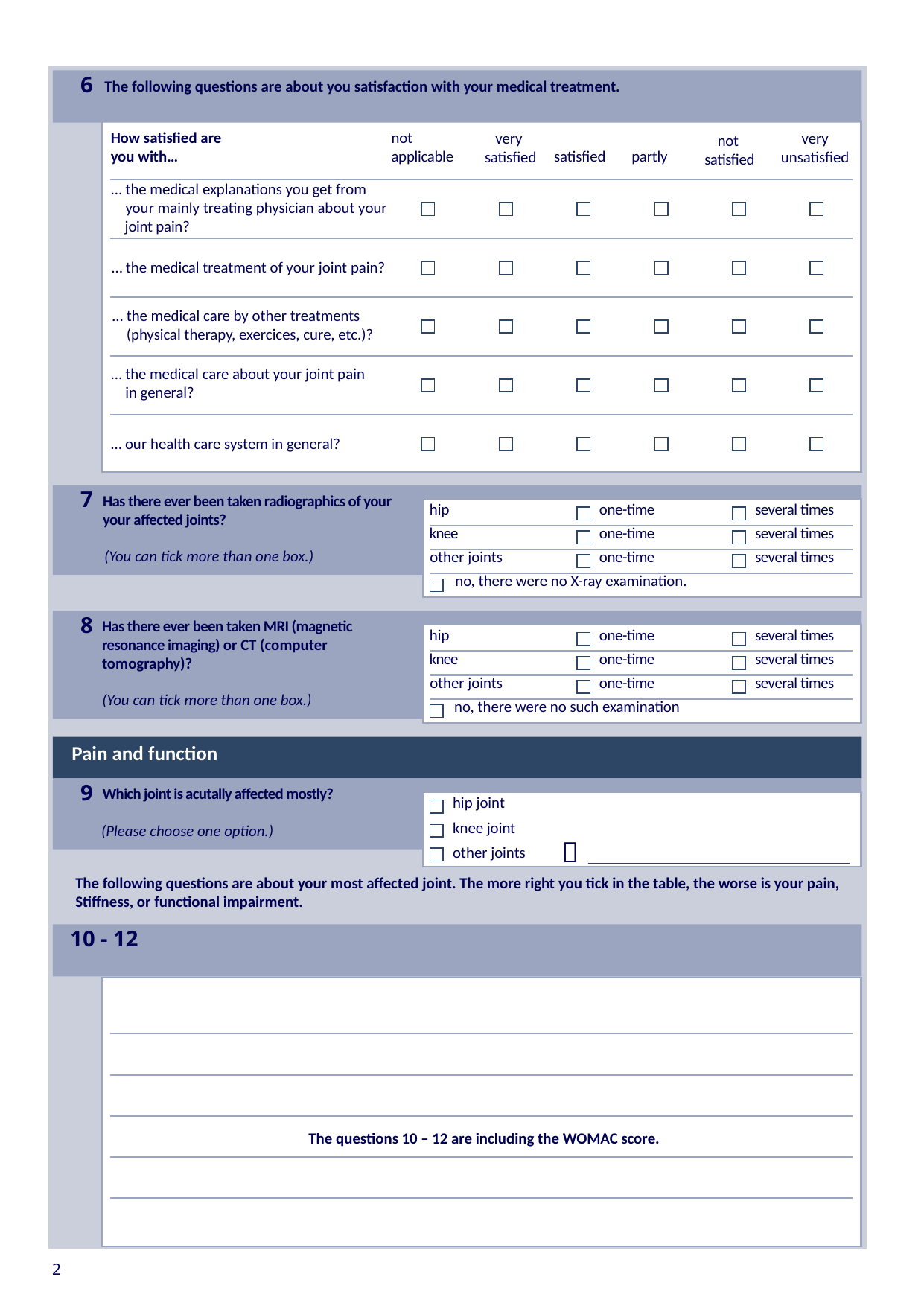

6
The following questions are about you satisfaction with your medical treatment.
How satisfied are
you with…
not
applicable
very
unsatisfied
very
satisfied
not
satisfied
satisfied
partly
… the medical explanations you get from
 your mainly treating physician about your
 joint pain?
… the medical treatment of your joint pain?
… the medical care by other treatments
 (physical therapy, exercices, cure, etc.)?
… the medical care about your joint pain
 in general?
… our health care system in general?
7
Has there ever been taken radiographics of your
your affected joints?
hip
one-time
several times
knee
one-time
several times
(You can tick more than one box.)
other joints
one-time
several times
no, there were no X-ray examination.
8
Has there ever been taken MRI (magnetic
resonance imaging) or CT (computer
tomography)?
hip
one-time
several times
knee
one-time
several times
other joints
one-time
several times
(You can tick more than one box.)
no, there were no such examination
Pain and function
9
Which joint is acutally affected mostly?
hip joint
knee joint
other joints
(Please choose one option.)

The following questions are about your most affected joint. The more right you tick in the table, the worse is your pain,
Stiffness, or functional impairment.
10 - 12
The questions 10 – 12 are including the WOMAC score.
2

## Slide 3
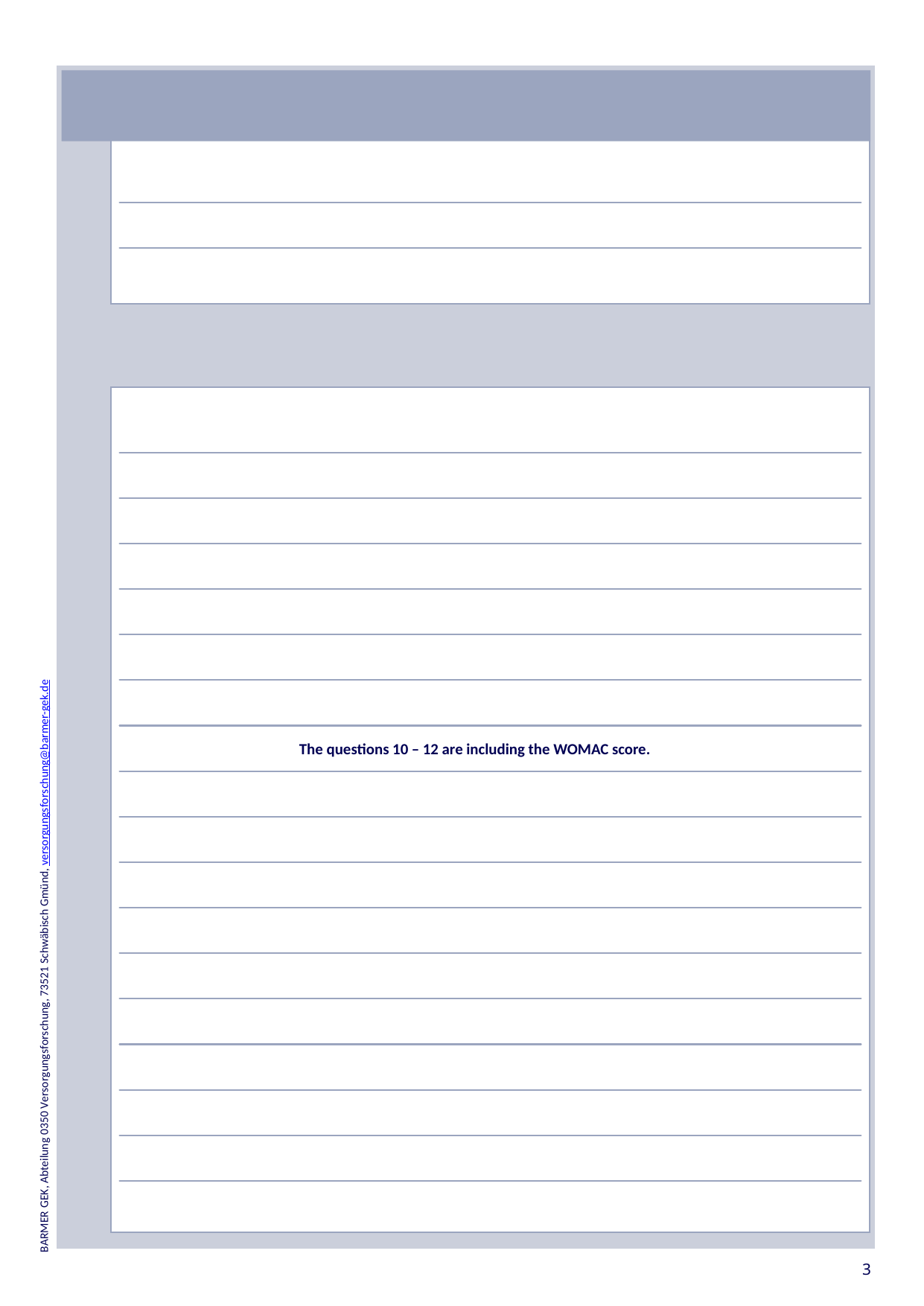

The questions 10 – 12 are including the WOMAC score.
BARMER GEK, Abteilung 0350 Versorgungsforschung, 73521 Schwäbisch Gmünd, versorgungsforschung@barmer-gek.de
3

## Slide 4
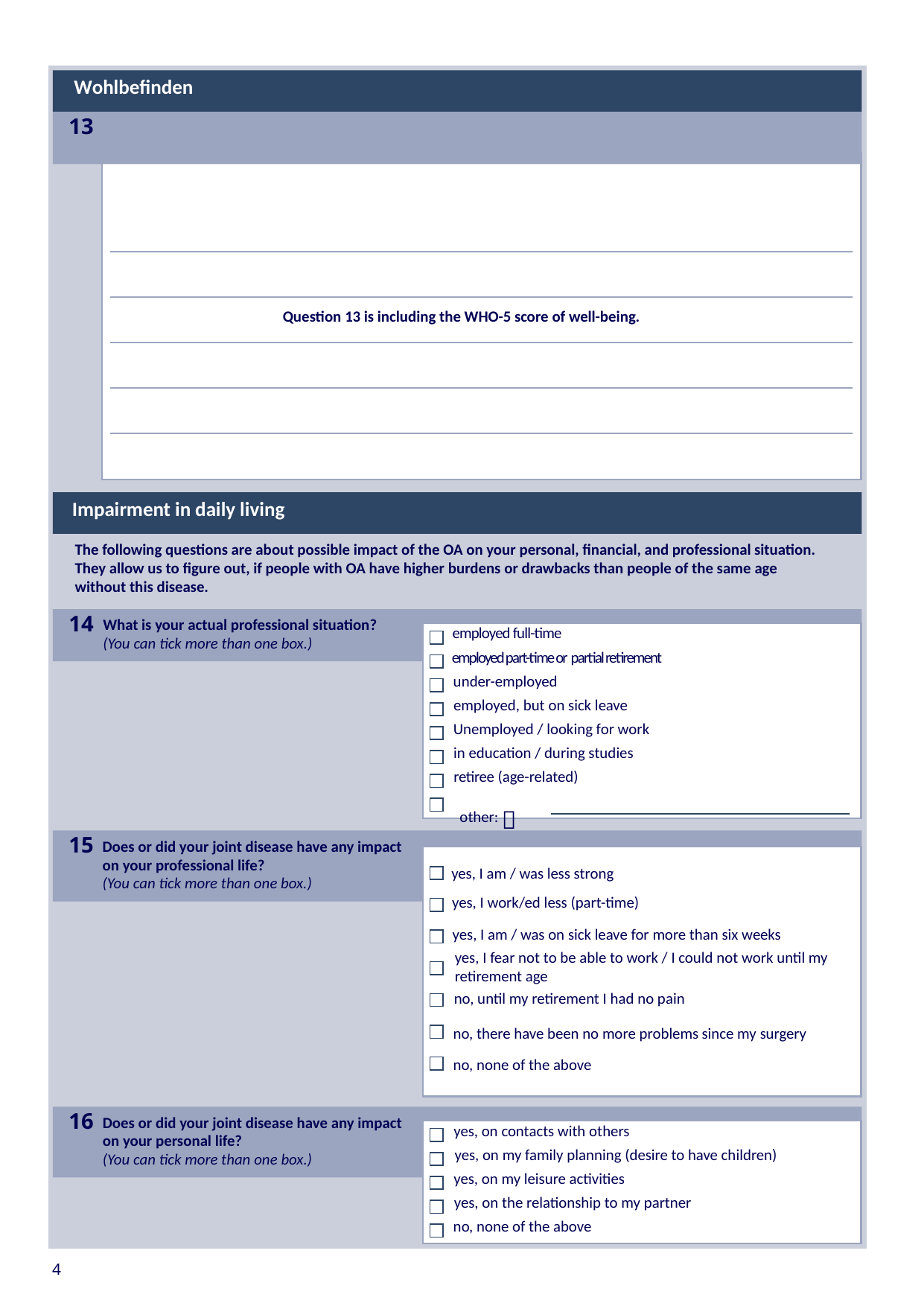

Wohlbefinden
13
Question 13 is including the WHO-5 score of well-being.
Impairment in daily living
The following questions are about possible impact of the OA on your personal, financial, and professional situation.
They allow us to figure out, if people with OA have higher burdens or drawbacks than people of the same age
without this disease.
14
What is your actual professional situation?
(You can tick more than one box.)
employed full-time
employed part-time or partial retirement
under-employed
employed, but on sick leave
Unemployed / looking for work
in education / during studies
retiree (age-related)
other:
15
Does or did your joint disease have any impact
on your professional life?
(You can tick more than one box.)
yes, I am / was less strong
yes, I work/ed less (part-time)
yes, I am / was on sick leave for more than six weeks
yes, I fear not to be able to work / I could not work until my
retirement age
no, until my retirement I had no pain
no, there have been no more problems since my surgery
no, none of the above
16
Does or did your joint disease have any impact
on your personal life?
(You can tick more than one box.)
yes, on contacts with others
yes, on my family planning (desire to have children)
yes, on my leisure activities
yes, on the relationship to my partner
no, none of the above
4

## Slide 5
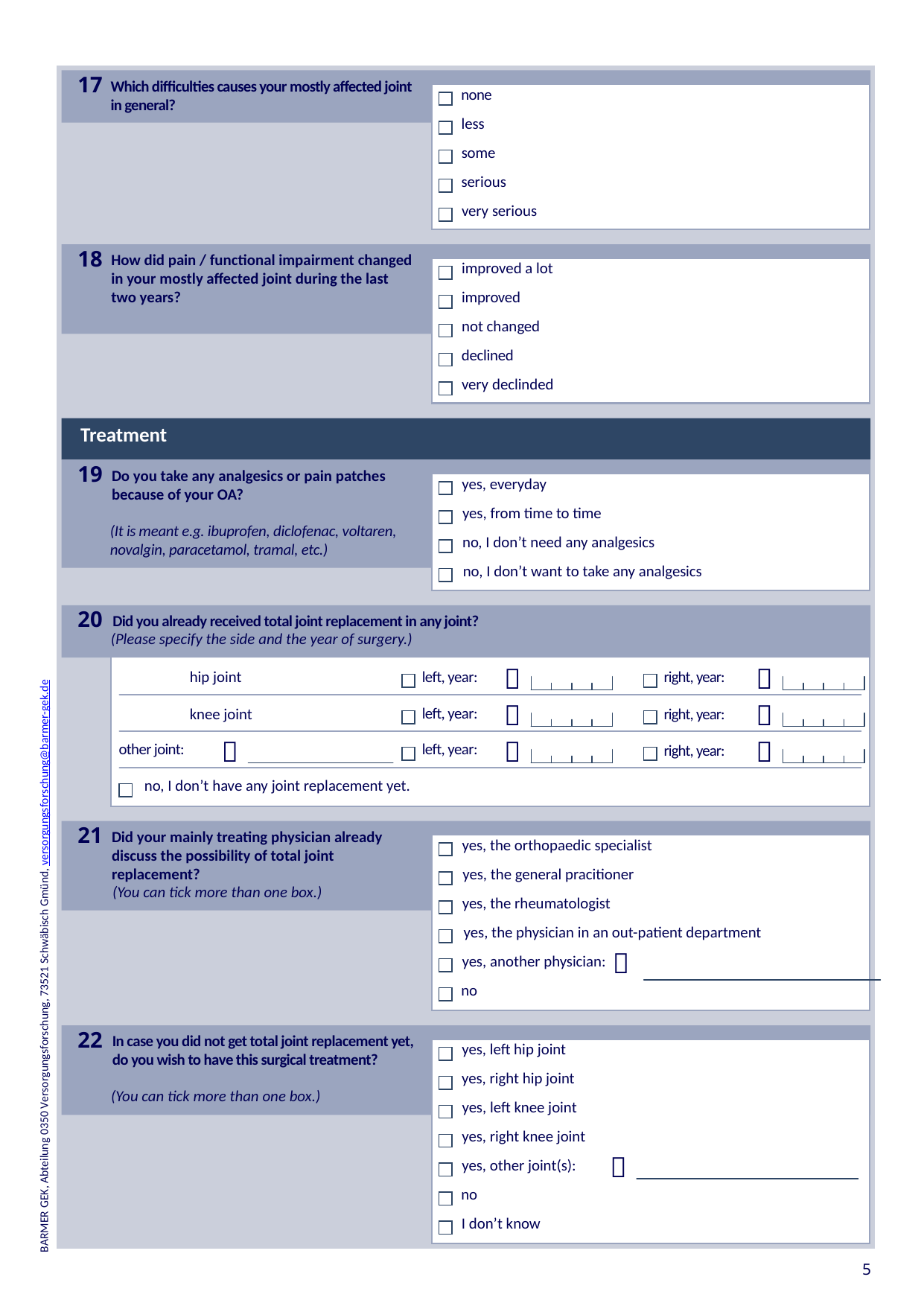

17
Which difficulties causes your mostly affected joint
in general?
none
less
some
serious
very serious
18
How did pain / functional impairment changed
in your mostly affected joint during the last
two years?
improved a lot
improved
not changed
declined
very declinded
Treatment
19
Do you take any analgesics or pain patches
because of your OA?
yes, everyday
yes, from time to time
(It is meant e.g. ibuprofen, diclofenac, voltaren,
novalgin, paracetamol, tramal, etc.)
no, I don’t need any analgesics
no, I don’t want to take any analgesics
20
Did you already received total joint replacement in any joint?
(Please specify the side and the year of surgery.)






left, year:
right, year:
right, year:
right, year:
hip joint
knee joint
left, year:

other joint:
left, year:
no, I don’t have any joint replacement yet.
21
Did your mainly treating physician already
discuss the possibility of total joint
replacement?
yes, the orthopaedic specialist
yes, the general pracitioner
(You can tick more than one box.)
yes, the rheumatologist
yes, the physician in an out-patient department

yes, another physician:
BARMER GEK, Abteilung 0350 Versorgungsforschung, 73521 Schwäbisch Gmünd, versorgungsforschung@barmer-gek.de
no
22
In case you did not get total joint replacement yet,
do you wish to have this surgical treatment?
yes, left hip joint
yes, right hip joint
(You can tick more than one box.)
yes, left knee joint
yes, right knee joint

yes, other joint(s):
no
I don’t know
5

## Slide 6
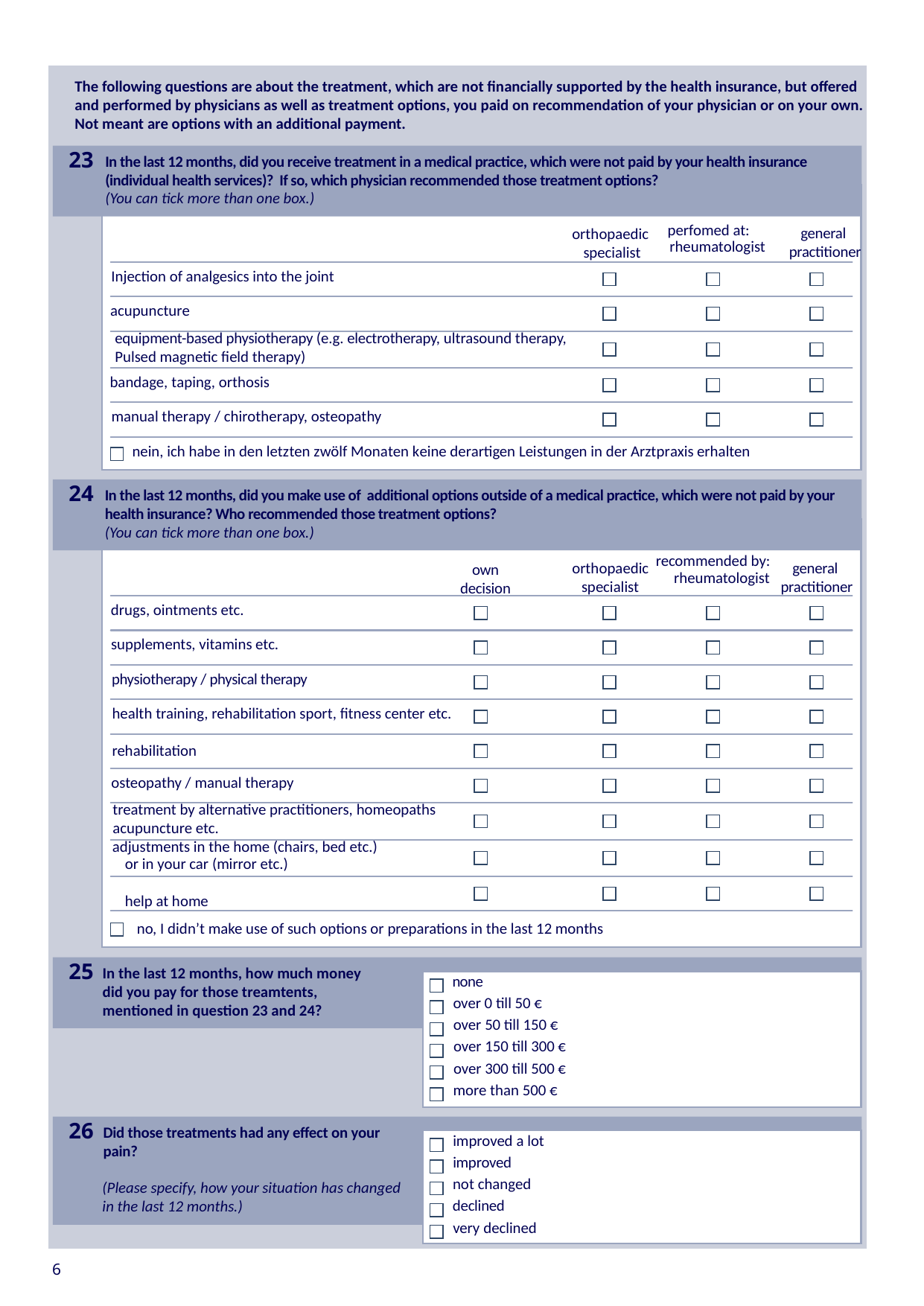

The following questions are about the treatment, which are not financially supported by the health insurance, but offered
and performed by physicians as well as treatment options, you paid on recommendation of your physician or on your own.
Not meant are options with an additional payment.
23
In the last 12 months, did you receive treatment in a medical practice, which were not paid by your health insurance
(individual health services)? If so, which physician recommended those treatment options?
(You can tick more than one box.)
perfomed at:
rheumatologist
general
practitioner
orthopaedic
specialist
Injection of analgesics into the joint
acupuncture
equipment-based physiotherapy (e.g. electrotherapy, ultrasound therapy,
Pulsed magnetic field therapy)
bandage, taping, orthosis
manual therapy / chirotherapy, osteopathy
nein, ich habe in den letzten zwölf Monaten keine derartigen Leistungen in der Arztpraxis erhalten
24
In the last 12 months, did you make use of additional options outside of a medical practice, which were not paid by your
health insurance? Who recommended those treatment options?
(You can tick more than one box.)
recommended by:
rheumatologist
general
practitioner
orthopaedic
specialist
own
decision
drugs, ointments etc.
supplements, vitamins etc.
physiotherapy / physical therapy
health training, rehabilitation sport, fitness center etc.
rehabilitation
osteopathy / manual therapy
treatment by alternative practitioners, homeopaths
acupuncture etc.
adjustments in the home (chairs, bed etc.)
or in your car (mirror etc.)
help at home
no, I didn’t make use of such options or preparations in the last 12 months
25
In the last 12 months, how much money
did you pay for those treamtents,
mentioned in question 23 and 24?
none
over 0 till 50 €
over 50 till 150 €
over 150 till 300 €
over 300 till 500 €
more than 500 €
26
Did those treatments had any effect on your
pain?
improved a lot
improved
not changed
(Please specify, how your situation has changed
in the last 12 months.)
declined
very declined
6

## Slide 7
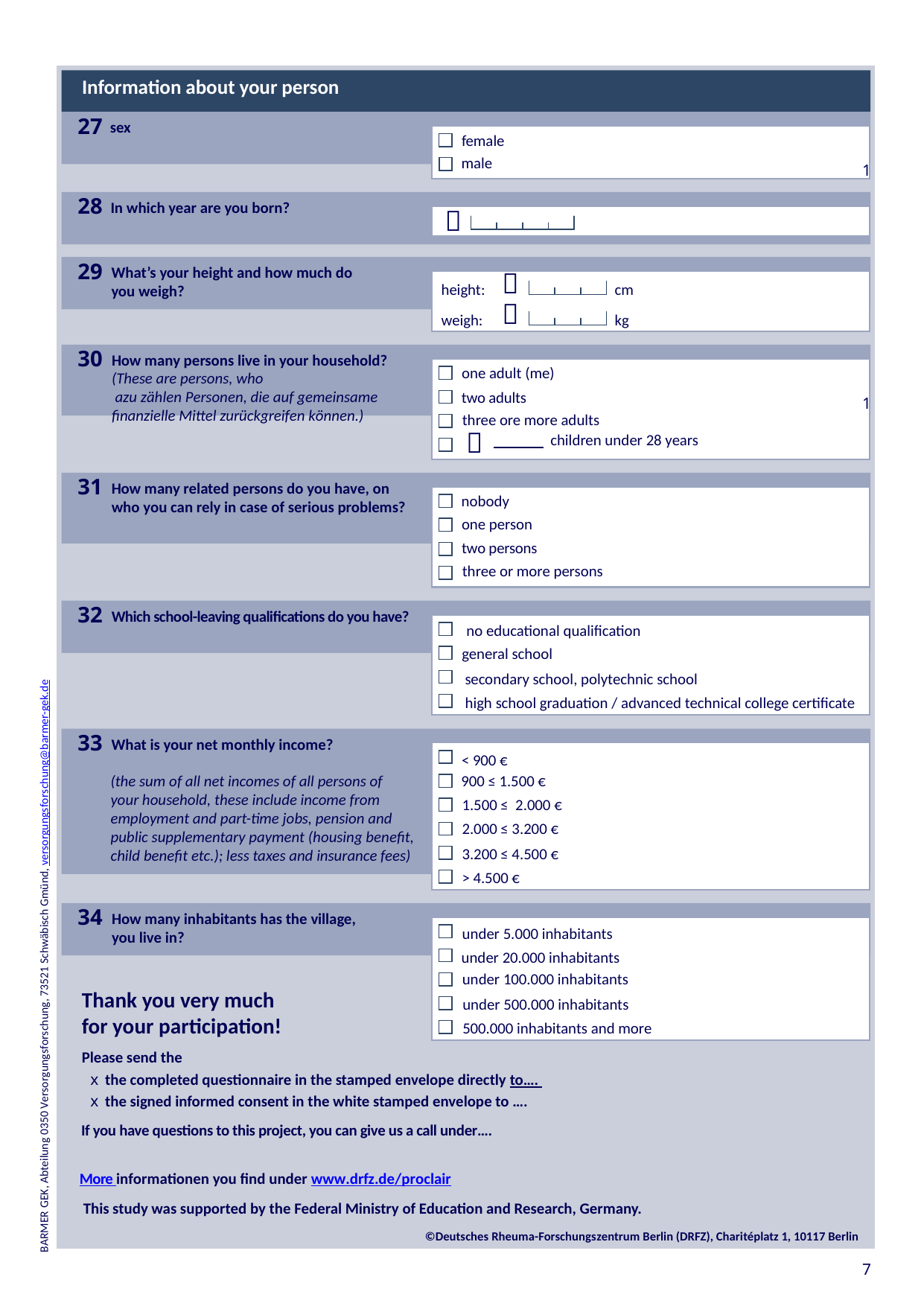

Information about your person
27
sex
female
male
1
28
In which year are you born?

29
What’s your height and how much do
you weigh?

height: 	cm
weigh: 	kg

30
How many persons live in your household?
(These are persons, who
 azu zählen Personen, die auf gemeinsame
finanzielle Mittel zurückgreifen können.)
one adult (me)
two adults
1
three ore more adults

 children under 28 years
31
How many related persons do you have, on
who you can rely in case of serious problems?
nobody
one person
two persons
three or more persons
32
Which school-leaving qualifications do you have?
no educational qualification
general school
secondary school, polytechnic school
high school graduation / advanced technical college certificate
33
What is your net monthly income?
< 900 €
(the sum of all net incomes of all persons of
your household, these include income from
employment and part-time jobs, pension and
public supplementary payment­ (housing benefit,
child benefit etc.); less taxes and insurance fees)
900 ≤ 1.500 €
1.500 ≤ 2.000 €
2.000 ≤ 3.200 €
3.200 ≤ 4.500 €
> 4.500 €
34
How many inhabitants has the village,
you live in?
under 5.000 inhabitants
under 20.000 inhabitants
BARMER GEK, Abteilung 0350 Versorgungsforschung, 73521 Schwäbisch Gmünd, versorgungsforschung@barmer-gek.de
under 100.000 inhabitants
Thank you very much
for your participation!
Please send the
under 500.000 inhabitants
500.000 inhabitants and more
x the completed questionnaire in the stamped envelope directly to….
x the signed informed consent in the white stamped envelope to ….
If you have questions to this project, you can give us a call under….
More informationen you find under www.drfz.de/proclair
This study was supported by the Federal Ministry of Education and Research, Germany.
©Deutsches Rheuma-Forschungszentrum Berlin (DRFZ), Charitéplatz 1, 10117 Berlin
7
